# Monitoring Avian Influenza A(H7N9) Virus through National Influenza-like Illness Surveillance, China

**DOI:** 10.3201/eid1908.130662

**Published:** 2013-08

**Authors:** Cuiling Xu, Fiona Havers, Lijie Wang, Tao Chen, Jinghong Shi, Dayan Wang, Jing Yang, Lei Yang, Marc-Alain Widdowson, Yuelong Shu

**Affiliations:** Chinese Center for Disease Control and Prevention, Beijing, China (C. Xu, L. Wang, T. Chen, J. Shi, D. Wang, J. Yang, L. Yang, Y. Shu);; US Centers for Disease Control and Prevention, Atlanta, Georgia, USA. (F. Havers, M.-A. Widdowson)

**Keywords:** influenza, avian influenza, avian influenza A(H7N9) virus, H7N9, subtype H7N9, China, influenza-like illness, surveillance, viruses

## Abstract

In China during March 4–April 28, 2013, avian influenza A(H7N9) virus testing was performed on 20,739 specimens from patients with influenza-like illness in 10 provinces with confirmed human cases: 6 (0.03%) were positive, and increased numbers of unsubtypeable influenza-positive specimens were not seen. Careful monitoring and rapid characterization of influenza A(H7N9) and other influenza viruses remain critical.

As of April 28, 2013, a total of 125 cases of avian influenza A(H7N9) virus infection and 24 related deaths were confirmed in humans in 8 provinces and 2 municipalities (hereafter called affected provinces/municipalities) of mainland China (*1*). The median age of patients was 63 years; most were male and had a history of exposure to live poultry (*2*). The first confirmed case was reported on March 31. On April 3, the Chinese Center for Disease Control and Prevention (China CDC) distributed primers and probes specific for avian influenza A(H7N9) virus to all national influenza surveillance network laboratories in China. To better understand the epidemiology, geographic spread, and clinical spectrum of this virus in China, we describe the Chinese National Influenza-Like Illness Surveillance Network (CNISN) and analyze data collected since March 4, 2013.

## The Study

**Figure 1:**
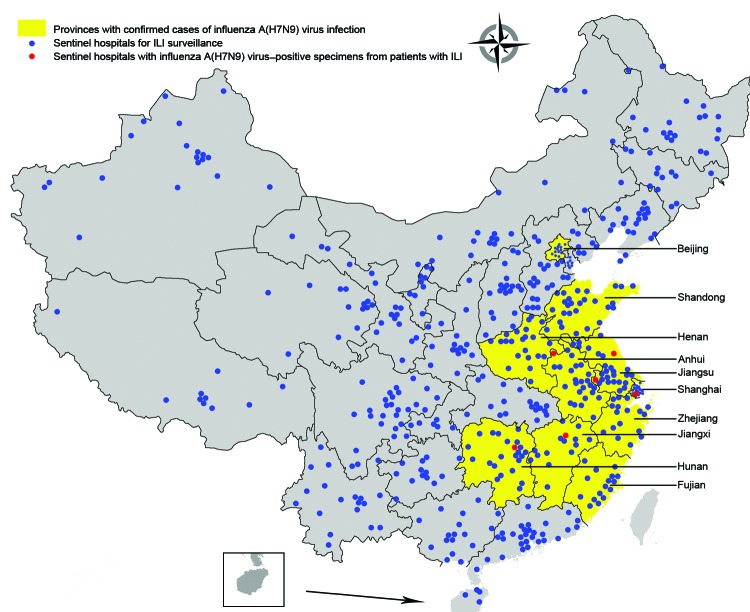
Geographic distribution of national influenza surveillance sentinel hospitals in Beijing and Shanghai Municipalities and 8 provinces with confirmed human cases of avian influenza A(H7N9) virus infection, China, 2013.

The CNISN includes 554 sentinel hospitals conducting surveillance for influenza-like illness (ILI; hereafter called sentinel hospitals) and 408 network laboratories in all 31 provinces of China (Figure 1). On a weekly basis, sentinel hospitals report the number of outpatient visits, by age group, for ILI and the total number of outpatients. Each week, 5–15 nasopharyngeal swab samples are collected from a convenience sample of patients who visit sentinel hospitals within 3 days of ILI onset. ILI is defined as temperature >38°C and cough or sore throat. Demographic and epidemiologic data, including age, sex, date of illness onset, and occupation, are also collected. Patient specimens are tested by real-time reverse transcription PCR or virus isolation in the affiliated laboratories.

On April 3, 2013, to enhance surveillance for influenza A(H7N9) virus, all network laboratories were required to increase the number of specimens to a minimum of 15/week and to test all specimens collected since March 4, 2013, for influenza A(H7N9) virus by real-time reverse transcription PCR as described (*3**,**4*). We analyzed data collected by CNISN during March 4–April 28. Population data by age group were provided by the National Bureau of Statistics of China.

During March 4–April 28, CNISN tested 46,807 nasopharyngeal swab samples from 554 sentinel hospitals throughout mainland China. Samples included 20,739 specimens from patients with ILI at 141 sentinel hospitals in 10 affected provinces/municipalities: Anhui, Jiangsu, Zhejiang, Shandong, Henan, Fujian, Jiangxi, and Hunan Provinces and Shanghai and Beijing Municipalities (Tables 1, 2). The median number of specimens collected each week from affected provinces/municipalities was 244 (range 72–792). Of the 20,739 samples from patients with ILI, 10,035 (48.4%) were from persons 0–14 years of age, 9,319 (44.9%) were from persons 15–59 years of age, and 1,385 (6.7%) were from persons >60 years of age. The age distribution of ILI cases in the 10 affected provinces/municipalities was substantially different from that in the overall population; persons 25–59 years of age had a lower proportion of ILI than would be expected had ILI distribution mirrored the age distribution of the population. (Technical Appendix, Figure 1). In the affected provinces/municipalities, the number of specimens tested increased from a mean of 2,643 during the week starting April 1 to a peak of 3,259 during the week starting April 9; the increase was highest among persons 15–24 and 25–59 years of age (Technical Appendix Figure 2).

**Table 1:** Number of ILI patients, by age, positive for avian influenza A(H7N9) virus, China, March 4–April 28, 2013*

Patient age, y	No. positive/no. tested	
	Persons from 10 outbreak-affected areas†	Persons from 21 non-affected provinces
0–4	2/6,333	0/10,419
5–14	0/3,702	0/4,452
15–24	0/3,210	0/3,259
25–59	3/6,109	0/6,627
>60	1/1,385	0/1,311
Total	6/20,739	0/26,068

*ILI, influenza-like illness. †Areas include Beijing and Shanghai Municipalities and Anhui, Jiangsu, Zhejiang, Shandong, Henan, Fujian, Jiangxi, and Hunan Provinces.

**Table 2:** Number of ILI patients positive for avian influenza A(H7N9) virus in areas with confirmed infections among humans, China, March 4–April 28, 2013*

Area†	No. positive/no. tested
Anhui	1/3,478
Beijing	0/1,392
Fujian	0/1,154
Henan	0/1,893
Hunan‡	1/1,912
Jiangsu	2/3,369
Jiangxi	1/1,588
Shandong	0/1,848
Shanghai	1/2,490
Zhejiang	0/1,615
Total	6/20,739

*ILI, influenza-like illness. †All areas are provinces, except Beijing Municipality. ‡Positive patient, a resident of Shanghai, stayed for 2–3 d in Hunan, where he visited the sentinel hospital and then returned to Shanghai.

**Figure 2:**
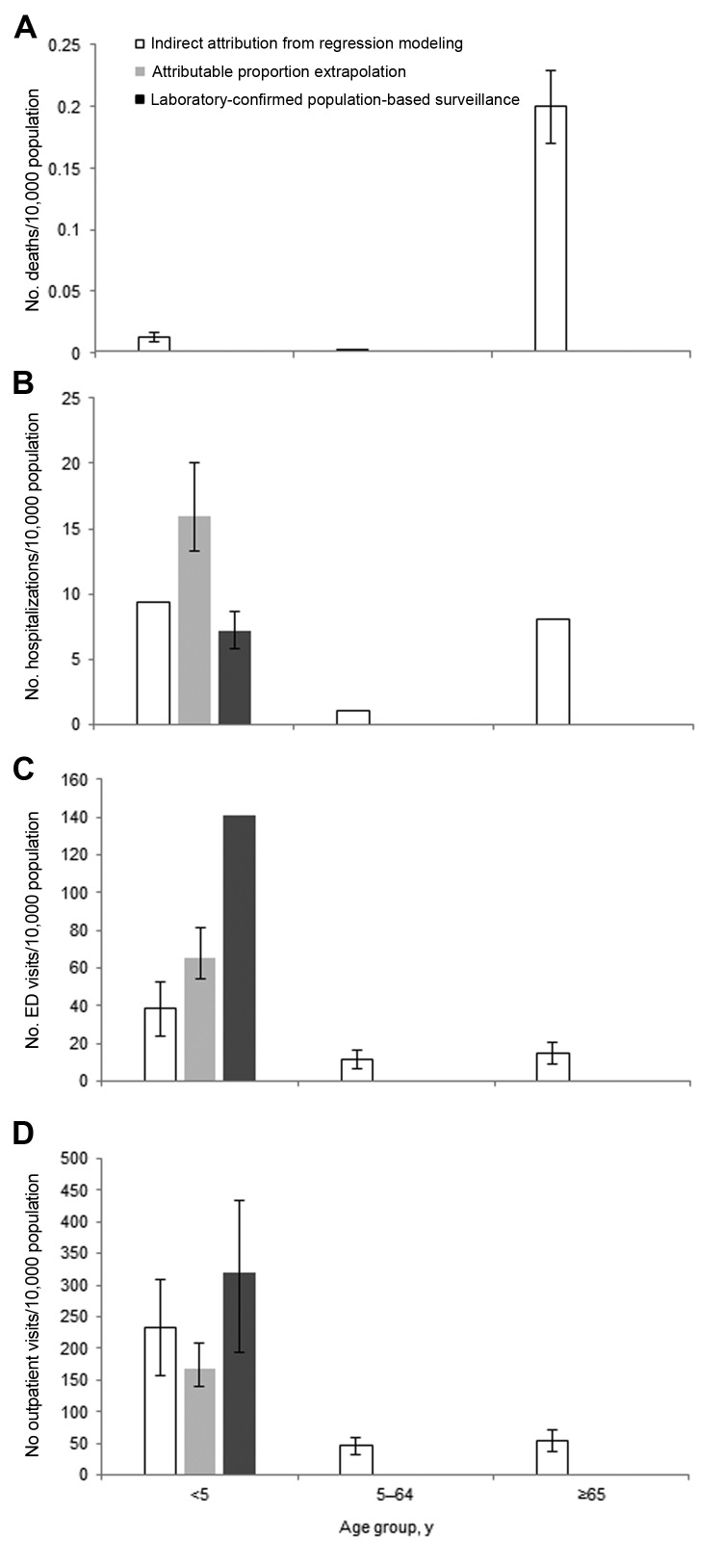
Percentage of hospital visits attributed to influenza-like illness, China, April 2, 2012–May 6, 2013. Hospital visits were made to sentinel surveillance hospitals in 7 southern provinces (SP) and 3 northern provinces/municipalities (NM, NP) with confirmed human cases of avian influenza A(H7N9) virus infection. Arrows indicate March 31, 2013, the date the first human case of influenza A(H7N9) virus infection was reported.

During April 1–28, the percentage of visits for ILI increased in 5 of the 7 affected southern provinces and 2 of 3 affected northern provinces/municipalities (Figure 2). However, during the same period, the proportion of specimens positive for influenza decreased in the affected provinces/municipalities.

Of the 10 affected provinces/municipalities, 5 reported >1 ILI patient with test results positive for influenza A(H7N9) virus. The percentage of specimens positive for influenza A(H7N9) virus, by province/municipality, ranged from 0 to 0.06% (Table 2). We detected influenza A(H7N9) virus in samples from 6 (0.03%) of the 20,739 patients with ILI; these cases were then reported as confirmed to the local CDCs and China CDC. No unsubtypeable influenza samples were reported in the affected provinces/municipalities during the study period (Technical Appendix Table).

Epidemiologic investigations found that 2 of the 6 patients with influenza A(H7N9) infection had not been hospitalized, and the other 4 had been hospitalized for pneumonia complications. The 2 patients who were not hospitalized were 2 and 4 years of age. Of the 4 hospitalized patients, 3 were 25–59 years of age, and 1 was 69 years of age. Four of the patients had a history of contact with live chickens or visiting a live poultry market.

## Conclusions

After the avian influenza A(H7N9) virus outbreak was identified in China, CNISN increased sampling and testing of ILI case-patients. CNISN has tested >46,807 specimens from all provinces, including 20,739 specimens from affected provinces/municipalities. As a result of this testing, CNISN identified 6 influenza A(H7N9) virus–positive specimens in 5 provinces that were already known to have cases. These data demonstrate that avian influenza A(H7N9) virus is an uncommon cause of ILI in any age group and in the areas reporting confirmed cases of influenza A(H7N9) infection. The confirmed case-patients included 2 children who did not require hospitalization and 4 adults with more severe disease, possibly indicating that influenza A(H7N9) virus causes milder disease in younger persons.

Although the proportion of all outpatient visits for ILI increased in affected provinces/municipalities, virologic surveillance data showed that the proportion of ILI patient specimens positive for influenza decreased, and there was no increase in unsubtypeable influenza viruses during the study period. This suggests that any increase in the percentage of consultations for ILI might be a result of increased healthcare–seeking behavior after media reports of the avian influenza A(H7N9) virus outbreak or the circulation of non-influenza respiratory viruses.

The spectrum of illness caused by other avian influenza viruses varies tremendously and can also vary by age group. Previous human infections with avian influenza A(H7) viruses (i.e., subtypes H7N3, H7N2, and H7N7) have been generally mild, causing conjunctivitis, with the exception of very occasional cases of pneumonia and a single fatal case in the Netherlands in a highly exposed veterinarian (*5*–*10*). In contrast, avian influenza A(H5N1) virus has an overall case fatality rate of 60%, and persons with confirmed cases are usually severely ill (*11*). Recent reviews of avian influenza A(H5N1) virus seroprevalence studies found little evidence that large numbers of human infections are going undetected (*12*–*14*). Among the 82 human influenza A(H7N9) virus infections reported as of April 17, 2013, a total of 38 (46%) were in persons >65 years of age (*2*). We did not find evidence of widespread mild disease, suggesting that the reported cases reflect the true distribution of infection and not a surveillance artifact.

Our study had several limitations. The 554 CNISN sentinel hospitals are located in urban areas, so the surveillance system may not detect influenza A(H7N9) virus infections in rural areas. In addition, most sentinel hospitals are tertiary care hospitals, and their patient populations are not representative of the general population with ILI. The distribution of those patients who had specimens tested is not necessarily random and may not reflect the population of those with ILI. Last, our system lacks a straightforward way to calculate rates of disease because it lacks denominators.

The emergence of a reassortant between avian influenza A(H7N9) virus and seasonal influenza subtype viruses, with possible increased human transmissibility, is possible during the upcoming summer influenza season in southern China. Careful monitoring and rapid characterization of influenza A(H7N9) viruses and unsubtypeable viruses from infected humans will be critical. Enhanced surveillance studies of mild and severe respiratory disease and seroprevalence studies in focal areas are necessary to further characterize the epidemiology and clinical spectrum of this emerging virus.

Technical Appendix Number of influenza virus–positive respiratory specimens, by virus type/subtype, in provinces with confirmed human influenza A(H7N9) virus infections; age distribution of patients seen for influenza-like illness in areas with confirmed human influenza A(H7N9) virus infections; and number of specimens tested for avian influenza A(H7N9) virus in 10 provinces/municipalities with confirmed human cases, China, March 4–April 28, 2013.
