# Supplementary material for: Monitoring Avian Influenza A(H7N9) Virus through National Influenza-like Illness Surveillance, China
Source: Emerg Infect Dis. 2013 Aug;19(8):1289–92. doi: 10.3201/eid1908.130662 (PMC3739526; doi:10.3201/eid1908.130662)
Supplement: Technical Appendix — Number of influenza virus–positive respiratory specimens, by virus type/subtype, in provinces with confirmed human influenza A(H7N9) virus infections; age distribution of patients seen for influenza-like illness in areas with confirmed human influenza A(H7N9) virus infections; and number of specimens tested for avian influenza A(H7N9) virus in 10 provinces/municipalities with confirmed human cases, China, March 4–April 28, 2013. [file 13-0662-Techapp-s1.pdf]

Article DOI: http://dx.doi.org/10.3201/eid1907.130662

Monitoring Avian Influenza A(H7N9) Virus through National Influenza-like Illness Surveillance, China

Technical Appendix

Technical Appendix Table. Number of influenza virus–positive respiratory specimens, by virus type/subtype, in 10 provinces affected by an outbreak of avian influenza A(H7N9) virus in humans, China, March 4–April 28, 2013

Patient age, y	Total no. positive for influenza/total no. tested (% positive)	No. positive (% of total) by influenza virus type					
		Influenza A					
		H1N1	H3N2	H1N1pdm	Unsubtypeable*	H7N9	Influenza B
0–4	487/6,333 (7.7)	0	80 (16.4)	396 (81.3)	0	2(0.4)	9 (0.4)
5–14	525/3,702 (14.2)	0	100 (19.0)	409 (77.9)	0	0	16 (0)
5–14	525/3,702 (14.2)	0	100 (19.0)	409 (77.9)	0	0	0
15–24	293/3,210(9.1)	0	62 (21.2)	217 (74.1)	0	0	14 (0)
25–59	551/6,109 (9.0)	0	109 (19.8)	424 (77)	0	3 (0.5)	15 (0.5)
≥60	101/1,385 (7.3)	0	21 (20.8)	73 (72.3)	0	1 (1.0)	6 (1.0)
Total	1957/20,739 (9.4)	0	372 (19.0)	1,519 (77.6)	0	6 (0.3)	45 (0.3)

*Specimens were positive for influenza A virus, but they could not be tested for subtypes (seasonal H1N1, H3N2, 2009 H1N1).

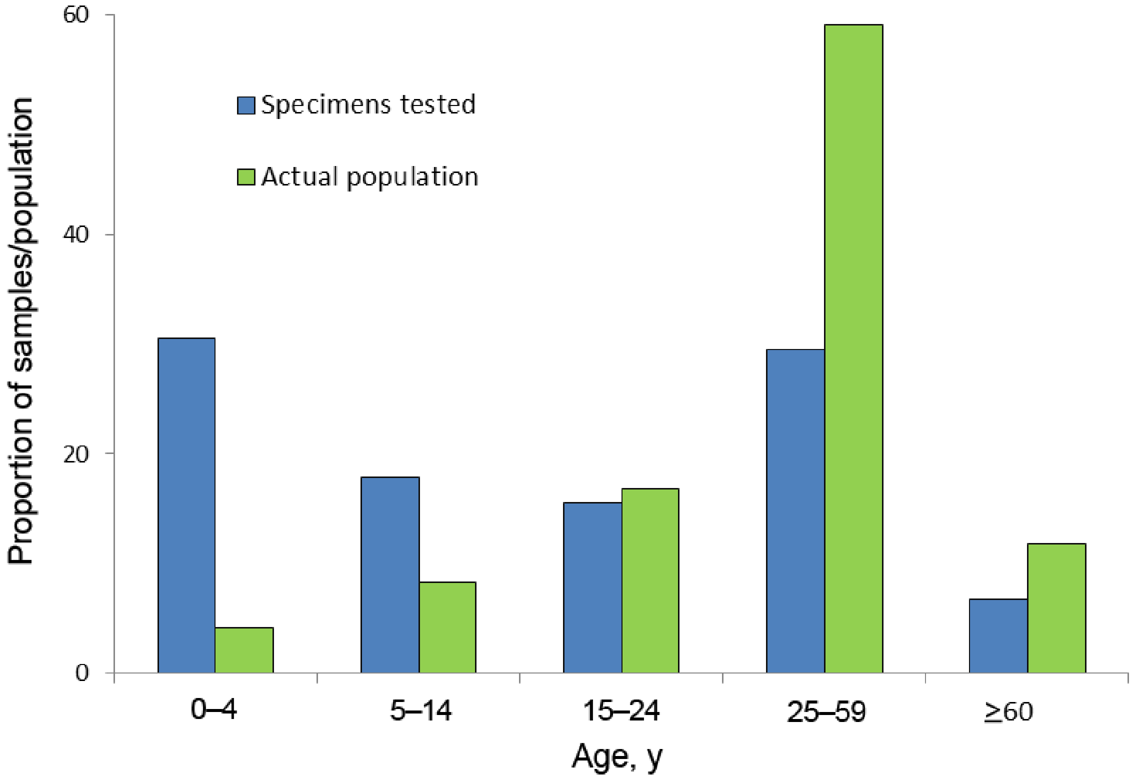

Technical Appendix Figure 1. Comparison of age distribution of the population in 10 municipality/provinces with confirmed human cases of avian influenza A(H7N9) virus infection and the age distribution of patients seen for influenza-like illness in sentinel surveillance hospitals in these areas, China, March 4–April 28, 2013.

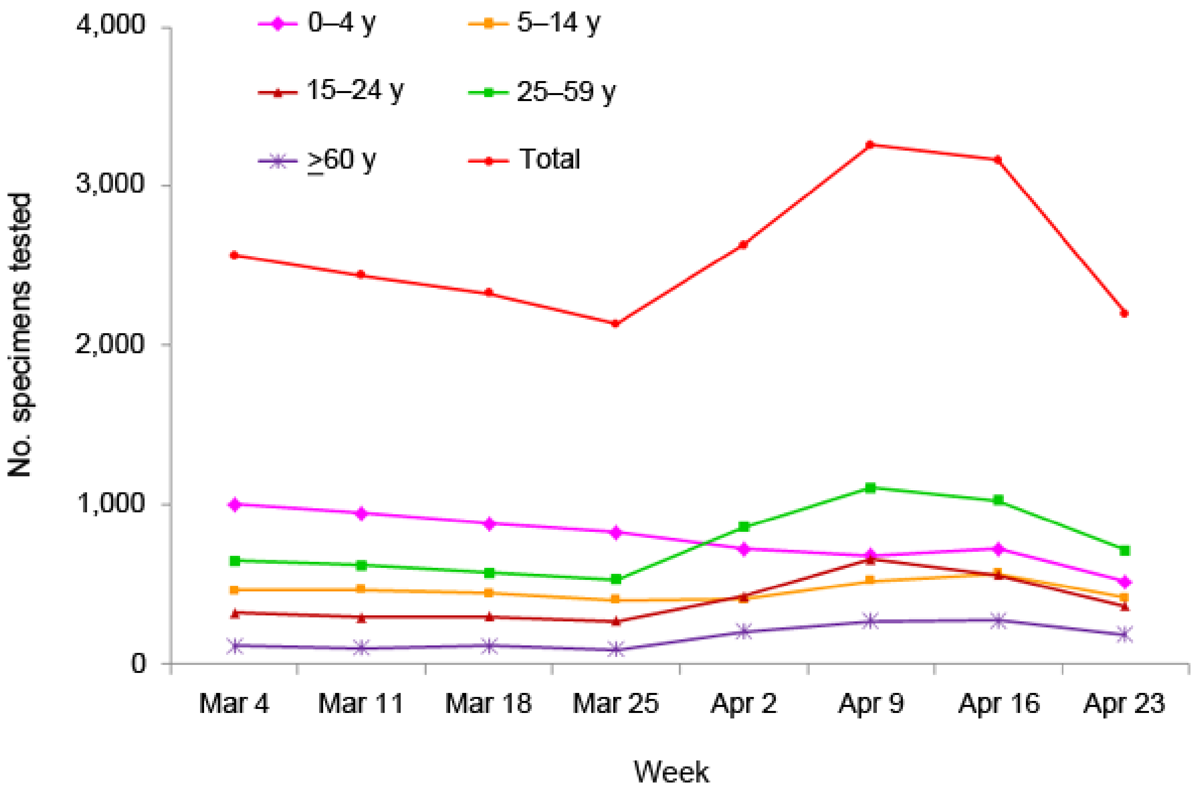

Technical Appendix Figure 2. Number of specimens tested for avian influenza A(H7N9) virus in 10 municipality/provinces with confirmed human cases, China, March 4–April 28, 2013. Samples were from patients seen for influenza-like illness at sentinel surveillance hospitals.
